# Diagnostic Accuracy of Dual Energy CT for Bone Marrow Edema of the Sacroiliac Joints Compared with MRI: A Systematic Review

**DOI:** 10.3390/jcm15093337

**Published:** 2026-04-27

**Authors:** Armando Perrella, Nunzia Di Meglio, Giulio Bagnacci, Paolo Tini, Giandomenico Roviello, Nicole Martini, Chiara Piscitello, Chiara Giraudo, Luca Cantarini, Bruno Frediani, Maria Antonietta Mazzei

**Affiliations:** 1 Department of Medical, Surgical and Neuro Sciences, University of Siena, 53100 Siena, Italy; 2 Unit of Diagnostic Imaging, Department of Medical Sciences, Azienda Ospedaliero-Universitaria Senese, 53100 Siena, Italy; 3 Unit of Radiation Oncology, Oncological Department, Azienda Ospedaliero-Universitaria Senese, 53100 Siena, Italy; 4 Department of Health Sciences, University of Florence, 50134 Florence, Italy; 5 Department of Cardiac, Thoracic, Vascular Sciences and Public Health—DCTV, University of Padova, 35122 Padova, Italy; 6 Rheumatology Unit, Department of Medical Sciences, Azienda Ospedaliero-Universitaria Senese, 53100 Siena, Italy

**Keywords:** Dual-Energy CT, virtual non-calcium, bone marrow edema, inflammatory sacroiliitis, axial spondyloarthritis, ankylosing spondylitis, meta-analysis, systematic review

## Abstract

**Background**: Dual-energy computed tomography (DECT) with virtual non-calcium (VNCa) reconstruction is emerging as an alternative method for identifying bone marrow edema (BME) in cases of inflammatory sacroiliitis, especially when magnetic resonance imaging (MRI) is contraindicated or unavailable. **Objectives**: The aim of this systematic review and meta-analysis is to evaluate the diagnostic performance of DECT with VNCa reconstruction for detecting BME in inflammatory sacroiliitis, using MRI as the reference standard. **Methods**: Following PRISMA-DTA guidelines and a pre-registered PROSPERO protocol (CRD420251103652), we searched PubMed, Web of Science, and the Cochrane Library up to June 2025. Seven studies comprising 358 patients and 591 sacroiliac joints were included. Quality assessment was performed using QUADAS-2. Pooled sensitivity and specificity were calculated using a bivariate random-effects regression model. Heterogeneity was evaluated using the I^2^ statistic. Subgroup analyses were performed for anatomical site and slice thickness, along with a meta-regression according to anatomical site. Publication bias was assessed using Deeks’ test. **Results**: Quality assessment revealed a moderate risk of bias, primarily related to patient selection. The pooled sensitivity and specificity of DECT VNCa were 78% (95% CI: 65–88%) and 83% (95% CI: 71–91%), respectively. The area under the SROC curve was 0.91 (95% CI: 0.88–0.93), indicating good diagnostic accuracy. Heterogeneity was moderate for sensitivity (I^2^ ≈ 68%) and high for specificity (I^2^ ≈ 88%), largely driven by one outlier study. Sensitivity was lower for sacral BME than for iliac BME (69% vs 79%), although this difference did not reach statistical significance (*p* ≈ 0.07). Specificity tended to be higher with slice thickness ≥1 mm than with <1 mm (90% vs 84%), although the difference was not statistically significant (*p* ≈ 0.06). Exclusion of one outlier study (Deppe et al.) increased pooled specificity to 92% and reduced heterogeneity. Deeks’ test did not reveal significant publication bias. **Conclusions**: DECT VNCa has been shown to be highly accurate in the diagnosis of inflammatory sacroiliac BME. Its high specificity makes it suitable for confirming inflammatory activity when an MRI scan is not feasible. However, its sensitivity is moderate and variable, with a trend towards lower values for sacral lesions. This precludes its use as a standalone test to rule out active inflammatory sacroiliitis, especially in young patients. Standardization of acquisition protocols and site-specific diagnostic thresholds is recommended for optimal clinical implementation. **Clinical Impact**: Clarifying the diagnostic performance of VNCa of DECT to define its role as a complementary tool to MRI for assessing BME in inflammatory sacroiliitis. These results could guide appropriate patient selection and highlight the need for standardized protocols.

## 1. Introduction

Inflammatory sacroiliitis, characterized by inflammatory changes including bone marrow edema (BME) of the sacroiliac joints (SIJs), represents a hallmark feature of axial spondyloarthritis (axSpA). Magnetic resonance imaging (MRI) with fluid-sensitive sequences (STIR or short tau inversion recovery and fat-saturated T2-weighted imaging) is currently the reference standard for detecting active inflammatory lesions due to its excellent soft tissue contrast and direct visualization of bone marrow lesions [1]. However, MRI presents some limitations, including cost, limited accessibility in certain settings, uncooperative patients and contraindications (e.g., certain metallic implants, claustrophobia), and relatively long acquisition times, which patients with active inflammatory pain may find difficult to tolerate.

Dual-energy computed tomography (DECT) is an emerging technique with several applications in different clinical scenarios [2,3,4]. The physical principle of DECT exploits the differential contribution of the photoelectric effect to X-ray absorption, allowing for material characterization based on energy-dependent attenuation profiles [5]. DECT with virtual non-calcium (VNCa) reconstruction has emerged as a promising alternative imaging modality for detecting BME: by leveraging differential X-ray attenuation properties at two distinct energy levels, DECT enables computational subtraction of trabecular bone, thereby highlighting the direct visualization of the underlying marrow attenuation [6]. Post-processing techniques include two- or three-material decomposition algorithms tailored to the specific DECT hardware (e.g., rapid kVp-switching, dual-source or dual layer). These approaches enable BME assessment through qualitative evaluation using color overlay maps and quantitative analysis, with concentrations typically expressed in mg/cm^3^ [6,7,8]. Advances such as blended material decomposition and optimized acquisition parameters have further improved diagnostic accuracy [9,10]. DECT with VNCa enhances the visualization of bone marrow lesions while maintaining the inherent advantages of CT, including rapid acquisition, widespread availability, superior spatial resolution, and the ability to simultaneously assess structural damage, such as erosions, fat metaplasia, nonbridging bone bud, sclerosis, and ankylosis [1,11,12]. Although DECT VNCa has been extensively evaluated for traumatic BME in peripheral joints and vertebral fractures, its diagnostic performance in inflammatory sacroiliitis is less well characterized.

This systematic review and meta-analysis, conducted according to a pre-registered PROSPERO protocol (CRD420251103652), aims to provide a comprehensive assessment of the diagnostic accuracy of DECT VNCa for detecting BME in sacroiliitis, identify sources of heterogeneity across studies, and formulate evidence-based recommendations for clinical implementation and future research directions.

## 2. Materials and Methods

### 2.1. Protocol and Registration

This review was conducted in accordance with the Preferred Reporting Items for Systematic Reviews and Meta-Analyses of Diagnostic Test Accuracy (PRISMA-DTA) statement. The review protocol was registered in PROSPERO (CRD420251103652). As Supplementary Material Table S1, you can find the PRISMA checklist.

### 2.2. Search Strategy

A comprehensive systematic literature search was performed in PubMed, Web of Science, and the Cochrane Library from database inception to 15 June 2025. The search strategy combined Medical Subject Headings (MeSH) terms and free-text words related to “Dual-Energy CT” AND (“Sacroiliac Joint” OR “Sacroiliitis” OR “Ankylosing Spondylitis” OR “Axial Spondyloarthritis”). No language restrictions were initially applied, though only studies with English full-text were ultimately included. Reference lists of all eligible articles and relevant review papers were hand-searched to identify additional studies. Any disagreement was resolved by consensus.

### 2.3. Eligibility Criteria

Inclusion Criteria:

(i) Study Design: Observational studies (prospective or retrospective) reporting diagnostic accuracy.

(ii) Population: Adults (>18 years) with suspected or established sacroiliitis undergoing both DECT and MRI of the SIJs.

(iii) Index Test: DECT with VNCa reconstructions.

(iv) Reference Standard: MRI of the SIJs using sequences sensitive to BME (STIR or T2-weighted fat-saturated).

(v) Outcome: Sufficient data to construct a 2 × 2 contingency table (true positives [TP], false positives [FP], false negatives [FN], true negatives [TN]) at either the patient or joint level.

Exclusion Criteria:

(i) Publication Type: Abstracts, case reports, editorials, letters, comments, animal studies, or phantom studies.

(ii) Data: Duplicate or overlapping datasets (the most comprehensive publication was retained).

(iii) Sample Size: Studies with fewer than 15 patients (or 30 SIJs).

(iv) Language: Non-English full-text articles.

(v) Methodology: Studies lacking essential details on DECT acquisition or reconstruction protocols.

### 2.4. Study Selection and Data Extraction

**Figure 1:**
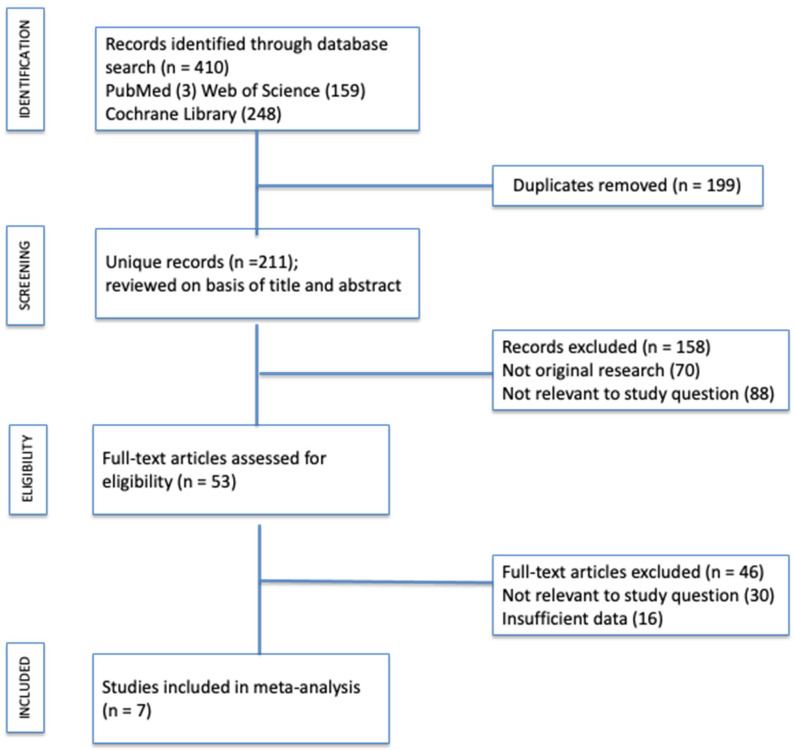
The PRISMA diagram shows the study selection process.

Two independent reviewers (A.P. and G.B., radiologists with 9 and 10 years of experience, respectively) screened titles and abstracts using Rayyan QCRI, a web-based software. Potentially relevant studies underwent full-text review. Disagreements were resolved by consensus or by consulting a third reviewer when necessary. A PRISMA flow diagram was created to document the selection process (Figure 1 and Figure S1). Data were independently extracted by the same two reviewers using a standardised, piloted form. Extracted data included study characteristics, patient demographics, imaging protocols, reader characteristics, and diagnostic performance metrics. Corresponding authors were contacted via email to request missing or unclear data.

### 2.5. Risk of Bias and Applicability Assessment

The methodological quality of each included study was independently assessed by two reviewers using the Quality Assessment of Diagnostic Accuracy Studies-2 (QUADAS-2) tool, evaluating four domains: patient selection, index test, reference standard, and flow/timing.

### 2.6. Statistical Analysis

Pooled estimates of sensitivity and specificity were calculated using a bivariate random-effects regression model, which accounts for the potential correlation between sensitivity and specificity as well as between-study heterogeneity. When multiple readers or analysis levels were reported, reader-averaged and study-level estimates were used when necessary. Diagnostic performance across the included studies was visualized using forest plots, with separate panels for sensitivity and specificity estimates. Each study’s point estimate was plotted alongside its 95% CI to assess data precision and distribution. Furthermore, a summary receiver operating characteristic (SROC) curve was generated based on the bivariate model parameters, and the area under the curve (AUC) was used to assess overall diagnostic performance. Between-study heterogeneity was evaluated using the I^2^ statistic for sensitivity and specificity. Potential sources of heterogeneity were explored through predefined subgroup analyses according to anatomical site (sacral vs. iliac BME) and slice thickness (<1 mm vs. ≥1 mm). Differences between subgroups were assessed using meta-regression analysis when possible. Sensitivity analyses were performed by sequentially excluding individual studies to assess the robustness of the pooled estimates. The certainty of evidence was assessed using the GRADE approach adapted for diagnostic test accuracy studies, incorporating QUADAS-2 assessments and evaluating risk of bias, inconsistency, indirectness, imprecision, and publication bias. Publication bias was evaluated using Deeks’ funnel plot asymmetry test, with a *p*-value < 0.10 indicating potential small-study effects. All statistical analyses were performed using STATA software (version 12.0; StataCorp, College Station, TX, USA) and Review Manager (RevMan, version 5.3; The Cochrane Collaboration). A two-sided *p*-value < 0.05 was considered statistically significant unless otherwise specified.

## 3. Results

### 3.1. Study Selection

**Figure 2:**
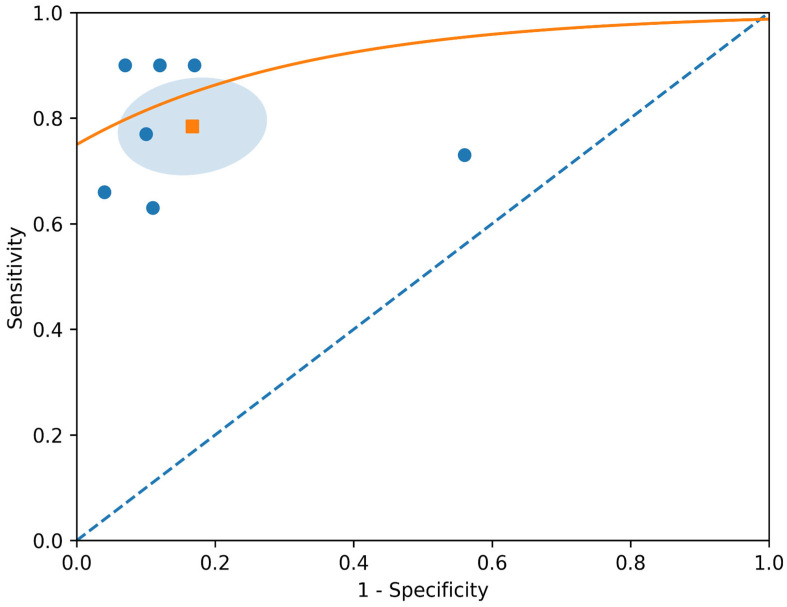
ROC curve in orange showing the diagnostic performance of DECT VNCa for detecting sacroiliac bone marrow edema (BME). Individual studies are represented as blue dots, and the summary estimate is shown as an orange square marker with its 95% confidence region (light blue circular area).

**Figure 3:**
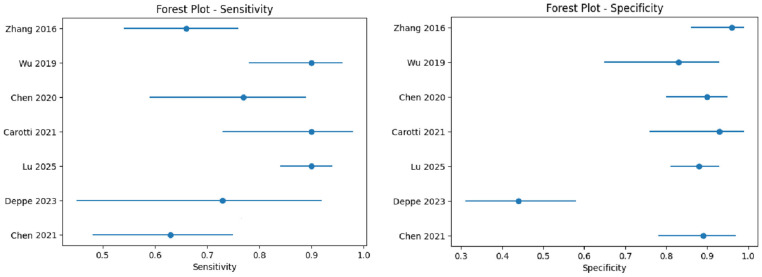
Forest plots of diagnostic performance across included studies. The left panel shows sensitivity estimates, and the right panel shows specificity estimates. Each point represents an individual study estimate, and horizontal lines indicate 95% confidence intervals. The outlier study (Deppe et al.) is clearly visible, particularly in the specificity analysis, contributing to the observed heterogeneity [13,14,15,16,17,18,19].

The initial database search yielded 410 records. After removing 199 duplicates, 211 unique records were screened by title and abstract. Of these, 53 articles underwent full-text review, with seven studies meeting all eligibility criteria for quantitative synthesis [8,9,10,11,12,13,14,15] (Figure 1). The characteristics of the seven included studies are summarized in Table 1 and Table 2. The studies were published between 2015 and 2025 and included a total of 358 patients and 591 SIJs. Four studies were prospective, and three were retrospective. DECT scanners included Siemens SOMATOM series (five studies), Canon Aquilion One (one study), and GE Discovery 750 HD (one study). Tube voltage combinations varied across studies (80/Sn150 kV, 100/Sn140 kV, 90/Sn150 kV), and slice thickness ranged from 0.6 to 2 mm. Sensitivity and specificity estimates of individual studies are presented in Table 3. The pooled sensitivity of DECT VNCa for detecting sacroiliac BME was 0.78 (95% CI: 0.65–0.88), with moderate heterogeneity (I^2^ ≈ 68%). The pooled specificity was 0.83 (95% CI: 0.71–0.91), with high heterogeneity (I^2^ ≈ 88%). The overall diagnostic performance was good, with a summary area under the ROC curve of 0.91 (95% CI: 0.88–0.93) (Figure 2). The forest plots revealed heterogeneity in diagnostic accuracy across the dataset (Figure 3): the study by Deppe et al. was identified as a clear outlier, particularly within the specificity analysis, where its estimate deviated significantly from the pooled trend of the other included studies.

### 3.2. Heterogeneity and Subgroup Analysis

Substantial heterogeneity was observed across studies, with moderate heterogeneity for sensitivity (I^2^ ≈ 68%) and high heterogeneity for specificity (I^2^ ≈ 88%). This variability was largely driven by one outlier study [18], which demonstrated markedly lower specificity (0.44) compared to the remaining studies. Sensitivity analysis excluding this study increased the pooled specificity to approximately 0.92 and reduced heterogeneity.

Subgroup analyses revealed consistent trends (Table 4):

(i) Anatomical site: sensitivity tended to be lower for sacral BME compared to iliac BME (approximately 69% vs. 79%); however, this difference did not reach statistical significance (*p* ≈ 0.07).

(ii) Technical parameters: analysis based on slice thickness showed a trend toward higher specificity for acquisitions ≥1 mm compared to <1 mm (approximately 90% vs. 84%), although this difference was not statistically significant (*p* ≈ 0.06).

The ROC curve (Figure 2) demonstrated good overall diagnostic performance, consistent with the pooled analysis (AUC ≈ 0.91). The confidence region around the summary estimate was relatively wide, reflecting substantial between-study heterogeneity. Deppe et al. was clearly identified as an outlier, lying outside the main cluster of studies due to its markedly lower specificity. Given the limited number of studies and variability in study design and reporting, subgroup comparisons should be interpreted with caution, and no statistically significant differences were confirmed.

### 3.3. Quality Assessment and Publication Bias

QUADAS-2 assessment revealed a moderate risk of bias, primarily in patient selection due to convenience sampling. The certainty of evidence was low for sensitivity and very low for specificity due to risk of bias and substantial heterogeneity across studies (Table S2). Concerns regarding applicability were low for the index and reference tests. Deeks’ funnel plot asymmetry test showed no significant publication bias (*p* = 0.24).

## 4. Discussion

The diagnostic gold standard for evaluating BME in inflammatory sacroiliitis remains MRI [1]. However, access to MRI may be limited in certain clinical settings, and the examination may be contraindicated in some patients. Conventional CT is unable to detect BME, representing its main limitation in this context. DECT has emerged as a promising technique that combines the advantages of CT, such as high spatial resolution, rapid acquisition, and widespread availability, with the ability to perform material decomposition. By exploiting differences in X-ray attenuation at two energy levels, DECT enables the generation of VNCa images, allowing indirect visualization of BME. Previous studies have reported good diagnostic performance of this technique, particularly in emergency settings [20,21,22,23,24,25,26]: overall, DECT demonstrates a pooled sensitivity of approximately 88–89% and a specificity of 96% compared with MRI, supporting its role as a rapid and accessible tool, particularly when MRI is unavailable [10,20].

From a clinical perspective, the results of this meta-analysis help better define the role of DECT with VNCa reconstruction in the diagnostic workflow for axSpA. While MRI remains the reference standard for comprehensive assessment of inflammatory and structural changes of the SIJs, DECT appears particularly suited as a complementary or problem-solving tool in selected clinical scenarios, especially when MRI is unavailable or contraindicated.

In the present analysis, sensitivity tended to be lower for sacral BME compared to iliac BME; however, this difference did not reach statistical significance (*p* ≈ 0.07). This trend may reflect anatomical and physiological differences between the two sites. The sacrum retains a higher proportion of red marrow into adulthood, which may reduce lesion conspicuity on VNCa images. In addition, the complex geometry of the sacral surface and susceptibility to beam-hardening artefacts may further limit detection. These factors suggest that DECT findings in the sacral region should be interpreted with caution, particularly in the presence of negative results, and necessitate site-specific diagnostic thresholds.

Interestingly, early axSpA involvement is thought to occur more frequently on the iliac side of the SIJs [27]. Several anatomical factors may contribute to this pattern. The iliac cartilage is thinner than the sacral cartilage [28], and the iliac side contains both hyaline and fibrocartilage, whereas the sacral side is composed predominantly of hyaline cartilage [29]. These structural differences may increase the susceptibility of the iliac side to inflammation, similar to the mechanisms hypothesized for enthesitis [30]. In addition, biomechanical factors, including load distribution across the SIJs, may further contribute to earlier inflammatory involvement of the iliac side [31].

Not only does the anatomical location of BME seem to affect the diagnostic performance, but as suggested by subgroup analyses, acquisition parameters and biological factors may play a role. A trend toward higher specificity was observed for slice thickness ≥ 1 mm, likely reflecting reduced noise and partial volume averaging effects, compared to thinner slices. However, this difference was not statistically significant (*p* ≈ 0.06) and should be interpreted with caution.

**Figure 4:**
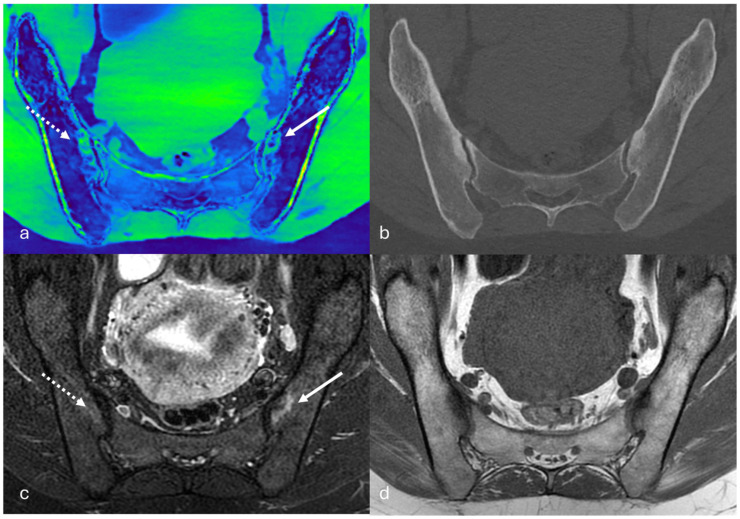
Comparison between DECT VNCa maps and MRI for BME detection. On the left iliac side, the BME identified on the VNCa map corresponds to the true BME seen on the fluid-sensitive MRI sequence (white arrow, (**a**,**c**)). Conversely, on the right iliac side, the high signal on the VNCa map does not correlate with true BME on MRI (dashed white arrow, (**a**,**c**)); instead, it represents an artefact caused by incomplete subtraction of subchondral sclerosis, which is clearly visible on both the bone-window CT (**b**) and the T1-weighted MRI sequence (**d**). The VNCa map was generated using a two-material decomposition algorithm from a spectral acquisition using an ultrafast-switching kVp CT system (Revolution Apex Elite, GE Healthcare, Milwaukee, WI, USA).

Subchondral sclerosis represents an important confounder, as it may interfere with material decomposition algorithms, especially in two-material decomposition algorithm, and reduce the accuracy of VNCa maps, particularly in chronic sacroiliitis, where inflammatory subchondral sclerosis frequently coexists with active inflammatory foci of BME, as well as in SIJs with degenerative sclerosis (Figure 4). In addition, differentiating inflammatory BME from physiological red marrow remains challenging, especially in younger patients and in anatomical regions where hematopoietic marrow persists. In such cases, MRI, particularly with diffusion-weighted imaging, remains essential for accurate tissue characterization [32].

A well-documented advantage of DECT is its enhanced sensitivity for BME in the elderly (≥60 years), which is attributed to age-related bone marrow conversion. The progressive increase in fatty marrow content with aging provides greater intrinsic contrast, making oedematous changes more conspicuous [20,21,22,23,24,25,26,27,28,29,30,31,33]. In line with the literature, we showed that thicker slices (≥1 mm) could present a better trend of diagnostic performance, likely because the increased image noise caused by thinner slices reduces image quality, and it is particularly relevant for the pelvis, where DECT is known to have a slightly poorer diagnostic performance [20].

From a clinical standpoint, the high specificity of DECT VNCa (0.83, 95% CI: 0.71–0.91) supports its use for confirming active inflammatory changes, particularly in adult patients with suspected disease exacerbation. However, its moderate sensitivity limits its role as a standalone tool to exclude active sacroiliitis, especially in younger patients with a high pre-test probability. In these cases, MRI remains indispensable. Importantly, reduced specificity in Deppe et al. (0.44) was observed even for the experienced reader, suggesting that factors beyond reader experience, including technical and methodological differences (low-dose CT, thin slice thickness), contributed to the outlier results. Furthermore, the low specificity estimate in the study by Deppe et al. [18] represents the main source of the substantial heterogeneity observed for overall specificity. These findings suggest that low-dose DECT failed to detect BME in patients with suspected axSpA, indicating that higher radiation doses may be required for DECT-based bone marrow analysis. Indeed, the low-dose protocol increases image noise—a factor not adequately compensated for by currently available post-processing software—and degrades bone marrow evaluation. This limits the effective separation of energy spectra necessary for the accurate identification and subtraction of calcium from the images.

Although DECT protocols maintain radiation dose comparable to conventional CT, radiation exposure represents a critical consideration, particularly in younger patients, especially females, and in patients requiring repeated imaging for disease follow-up or to evaluate response to treatment. Therefore, DECT should be used cautiously and primarily reserved for cases in which MRI is contraindicated or unavailable. Nevertheless, ongoing technological advances, including iterative reconstruction techniques, may help reduce radiation dose while maintaining or improving image quality.

The high specificity of DECT VNCa makes it particularly valuable for identifying active inflammatory change in chronic sacroiliitis, especially in adult patients experiencing a clinical exacerbation of inflammatory low-back pain. However, its moderate sensitivity means it should not be used to “rule out” inflammatory activity in young patients with high pre-test probability, related to the clinical-anamnestic-laboratory scenario of patients.

Notably, none of the studies reviewed to date has accounted for the spectral separation provided by photon-counting detector CT (PCD-CT). As a future perspective, PCD-CT represents a promising advancement for BME imaging, owing to its intrinsic spectral capabilities to generate VNCa images [34]. By directly counting photons and measuring their energy, PCD-CT provides higher spatial resolution, an improved contrast-to-noise ratio, and a reduced radiation dose compared with conventional energy-integrating detector CT [35,36,37]. Furthermore, its enhanced material decomposition accuracy may further improve BME detection and quantification, potentially approaching or complementing MRI performance in clinical practice [38].

This study has several limitations. The number of included studies was relatively small, and substantial heterogeneity was observed, particularly for specificity, likely reflecting differences in acquisition protocols and patient populations. While the hierarchical nature of the reported data (patient-, joint-, and quadrant-level) may introduce clustering-related bias, the lack of direct individual patient-level data precluded an explicit statistical correction for within-subject correlation, leading to an underestimation of the standard error and an inflation of apparent precision. Subgroup analyses were based on a limited number of studies and should therefore be interpreted with caution. Finally, most studies were conducted in specialized centers with experienced readers, which may limit generalizability to routine clinical practice.

## 5. Conclusions

DECT with VNCa reconstruction demonstrates good diagnostic accuracy for detecting sacroiliac BME, with high specificity (~0.83) supporting its role in confirming inflammatory activity when MRI is contraindicated or unavailable. However, moderate and variable sensitivity (~0.78), with a trend toward lower performance in sacral lesions, precludes its use as a standalone replacement for MRI in excluding active sacroiliitis, particularly in young patients. DECT may assume an increasingly important role in the management of axSpA, especially in selected clinical scenarios where MRI is not feasible. However, based on current evidence, MRI remains the reference standard for comprehensive assessment of sacroiliac inflammation, while DECT serves as a complementary tool with specific clinical applications. The substantial heterogeneity observed among included studies further highlights the need for protocol standardization. Differences in scanner technology, acquisition parameters, reconstruction algorithms, and slice thickness significantly influence diagnostic performance and limit comparability across studies. Future research should focus on establishing standardized DECT acquisition protocols and site-specific diagnostic thresholds, including differentiation between iliac and sacral BME. In addition, the integration of artificial intelligence-based post-processing tools may improve lesion detection and reduce reader dependency. Further prospective studies are needed.

## Figures and Tables

**Table 1:** Clinical characteristics of included studies.

First Author	Country	Patients (n)	SIJs/Units Analyzed (n)	Patients Mean Age (y)	Patients Sex Male (M) Female (F)	Reference Standard
Zhang et al. (2016) [13]	China	76	76	26.4	59 M 17 F	MRI
Wu et al. (2019) [14]	China	47	89	27	28 M 19 F	MRI
Chen M et al. (2020) [15]	China	40	80 (320 quadrants)	37.1	16 M 24 F	MRI
Carotti et al. (2021) [16]	Italy	40 (18)	36	48.6	30 M 10 F	MRI
Lu et al. (2025) [17]	China	42	84 (iliac sacral facet)	32.1	25 M 17 F	MRI
Deppe et al. (2023) [18]	Germany	68	136 (×24 quadrants)	40.2	40 M 28 F	MRI
Chen D et al. (2021) [19]	China	45	90 (180 quadrants)	34	21 M 24 F	MRI

**Table 2:** Study characteristics of included articles.

First Author	Study Design	Number of Readers	Reader Experience (y)	DECT Scanner	Slice Thickness (mm)	Reference Standard	k
Zhang et al. (2016) [13]	Retrospective	2	>5 y	GE Discovery 750 HD	0.625	1.5T MRI Signa HDxt GE	Not indicated
Wu et al. (2019) [14]	Prospective	2	17 y and 25 y	Siemens Somatom Definition Flash	1.0	3.0T MRI Ingenia Philips	0.81
Chen M et al. (2020) [15]	Prospective	2	4 y and 16 y	Siemens Somatom Definition Flash	1.0	3.0T MRI Prisma Siemens	0.7
Carotti et al. (2021) [16]	Prospective	2	20 y and 5 y	Siemens Somatom Force	1.0	1.5T MRI Achieva Philips	0.815
Lu et al. (2025) [17]	Prospective	2	>10 y for both readers	Siemens Third Generation DECT	1.0	1.5T MRI Philips	>0.61
Deppe et al. (2023) [18]	Retrospective	2	13 y and 3 y	Canon Aquilion One	0.6	1.5T MRI	**≤0.32**
Chen D et al. (2021) [19]	Retrospective	2	Not indicated	Siemens Somatom Definition Force	2	3.0T MRI Siemens Skyra	Not indicated

**Table 3:** Study-level sensitivity and specificity of included studies.

Study	Sensitivity (95% CI)	Specificity (95% CI)
Zhang et al. [13]	0.66 [0.54–0.76]	0.96 [0.86–0.99]
Wu et al. [14]	0.90 [0.78–0.96]	0.83 [0.65–0.93]
Chen M et al. [15]	0.77 [0.59–0.89]	0.90 [0.80–0.95]
Carotti et al. [16]	0.90 [0.73–0.98]	0.93 [0.76–0.99]
Lu et al. [17]	0.90 [0.84–0.94]	0.88 [0.81–0.93]
Deppe et al. [18]	0.73 [0.45–0.92]	0.44 [0.31–0.58]
Chen D et al. [19]	0.63 [0.48–0.75]	0.89 [0.78–0.97]

**Table 4:** Subgroup Analyses.

Subgroup	Studies	Overall Sensitivity (95% CI)	Overall Specificity (95% CI)	*p*-Value
**Slice Thickness**				
≥1 mm	Chen M et al. [15] Wu et al. [14] Carotti et al. [16] Lu et al. [17] Chen D et al. [19]	0.84 (0.74–0.91)	0.90 (0.86–0.93)	>0.05
<1 mm	Zhang et al. [13] Deppe et al. [18]	0.70 (0.55–0.85)	0.84 (0.76–0.86)	
**Anatomical Site**				
Ilium	Chen M et al. [15] Lu et al. [17] Chen D et al. [19]	0.79 (0.73–0.89)	0.89 (0.83–0.93)	>0.05
Sacrum		0.69 (0.58–0.72)	0.90 (0.86–0.94)	

## Data Availability

The data presented in this study are available on request from the corresponding author.

## Supplementary Materials

The following supporting information can be downloaded at: https://www.mdpi.com/article/10.3390/jcm15093337/s1, Table S1: PRISMA checklist: PRISMA 2020 for Abstract Checklist [39]; Table S2: Certainty of evidence was downgraded due to methodological limitations (QUADAS-2 assessment indicating moderate risk of bias, mainly in patient selection) and substantial heterogeneity across studies; Figure S1: The original PRISMA diagram shows the study selection process.

## Abbreviations

BME	Bone Marrow Edema
VNCa	Virtual Non-Calcium images
axSpA	Axial Spondyloarthritis
DECT	Dual-Energy Computed Tomography
SIJs	Sacroiliac Joints
